# Working Memory Maintenance of Visual and Auditory Spatial Information Relies on Supramodal Neural Codes in the Dorsal Frontoparietal Cortex

**DOI:** 10.3390/brainsci14020123

**Published:** 2024-01-24

**Authors:** Aurora Rizza, Tiziana Pedale, Serena Mastroberardino, Marta Olivetti Belardinelli, Rob H. J. Van der Lubbe, Charles Spence, Valerio Santangelo

**Affiliations:** 1 Department of Psychology, Sapienza University of Rome, P.le A. Moro 5, 00185 Rome, Italy; aurorarizza@gmail.com (A.R.); marta.olivetti@uniroma1.it (M.O.B.); 2 Functional Neuroimaging Laboratory, IRCCS Santa Lucia Foundation, Via Ardeatina 306, 00179 Rome, Italy; tiziana.pedale@gmail.com; 3 Department of Philosophy, Social Sciences & Education, University of Perugia, Piazza G. Ermini 1, 06123 Perugia, Italy; serena.mastroberardino@unipg.it; 4 ECONA, Interuniversity Centre for Research on Cognitive Processing in Natural and Artificial Systems, Sapienza University of Rome, Via dei Marsi 78, 00185 Rome, Italy; 5 Cognition, Data and Education, University of Twente, Drienerlolaan 5, 7522 NB Enschede, The Netherlands; r.h.j.vanderlubbe@utwente.nl; 6 Laboratory of Vision Science and Optometry, Adam Mickiewicz University, Wieniawskiego 1, 61-712 Poznan, Poland; 7 Department of Experimental Psychology, University of Oxford, Anna Watts Building, Oxford OX2 6BW, UK; charles.spence@psy.ox.ac.uk

**Keywords:** visual, auditory, spatial, working memory, fMRI

## Abstract

The frontoparietal attention network plays a pivotal role during working memory (WM) maintenance, especially under high-load conditions. Nevertheless, there is ongoing debate regarding whether this network relies on supramodal or modality-specific neural signatures. In this study, we used multi-voxel pattern analysis (MVPA) to evaluate the neural representation of visual versus auditory information during WM maintenance. During fMRI scanning, participants maintained small or large spatial configurations (low- or high-load trials) of either colour shades or sound pitches in WM for later retrieval. Participants were less accurate in retrieving high- vs. low-load trials, demonstrating an effective manipulation of WM load, irrespective of the sensory modality. The frontoparietal regions involved in maintaining high- vs. low-load spatial maps in either sensory modality were highlighted using a conjunction analysis. Widespread activity was found across the dorsal frontoparietal network, peaking on the frontal eye fields and the superior parietal lobule, bilaterally. Within these regions, MVPAs were performed to quantify the pattern of distinctness of visual vs. auditory neural codes during WM maintenance. These analyses failed to reveal distinguishable patterns in the dorsal frontoparietal regions, thus providing support for a common, supramodal neural code associated with the retention of either visual or auditory spatial configurations.

## 1. Introduction

Working memory (WM) is the cognitive process that enables information to be maintained in an easy-to-access state for a short time period [1,2]. A conventional approach for probing the neural mechanisms implicated in various WM stages is the delayed match-to-sample task [3]. This task enables researchers to assess the encoding of the memorandum, the maintenance of the previously presented material, and the recognition of whether the test stimulus is part or not of the encoded memorandum. Many WM studies have focused on the neural correlates of the maintenance phase (see [4], for a review). These studies have consistently suggested a pivotal role played by the frontoparietal attention network [5,6,7,8,9], whose activation was found to increase as a function of the number of items to be retained in WM (i.e., in conditions of increased WM load [6,10,11,12,13,14]). Moreover, the frontoparietal network also appears to be involved in shifting attention from the externally presented test stimulus to the internal representation of the stimulus to be maintained in memory [15,16,17]. The involvement of these regions is therefore thought to reflect attention-based mechanisms, which are required to sustain the maintenance of WM information (e.g., [18,19,20,21]). However, it is still a subject of debate whether the contribution of this network is supramodal, involving the same pattern of brain activation regardless of the stimulus modality, or whether it involves modality-specific activation patterns (see [22,23], for reviews).

Some evidence indicates that frontoparietal regions process information in a “supramodal” manner (e.g., [24,25,26,27,28,29]). In contrast, other studies have found modality-specific activations within the frontoparietal attentional network (see, e.g., [30,31,32,33]). For example, two fMRI studies explored this issue by using two comparable versions of the n-back task, either with visual or auditory stimuli [34,35]. Both studies reported different frontoparietal activations between the two task modalities, namely, increased activity in the posterior parietal cortex for the visual n-back and increased activity in the dorsolateral prefrontal cortex for the auditory n-back (though see [36] for a PET study revealing common activations for visual and auditory n-back tasks). However, it is worth noting that in these studies, brain activations were recorded during visual and auditory stimulus presentation (i.e., not during stimulus maintenance). As far as we know, only Majerus and colleagues [20] have explored the specialization of frontoparietal regions during the WM maintenance phase, that is, in the absence of any sensory stimulation. In this study, they presented two separate delayed match-to-sample tasks with visual (coloured squares) and verbal (written) stimuli, presented at one of three load conditions (low, medium, and high). Specifically for the visual task, arrays of 2, 4, or 6 coloured squares were presented during the encoding phase, whereas for the verbal task, arrays of 2, 4, or 6 written consonant letters were presented. Multi-voxel pattern analysis (MVPA) was used to decode the neural patterns in the frontoparietal network that predicted high vs. low load (i.e., 2 vs. 6 to-be-maintained items) in each of the two task modalities, visual and verbal. A classifier was trained to distinguish the neural patterns associated with high vs. low WM load in one modality and tested the ability of that classifier to predict the WM load in the other modality, and vice versa, during all WM phases, namely, encoding, maintenance, and retrieval. Majerus and colleagues reported high decoding accuracy of WM load in the frontoparietal regions, especially during encoding and maintenance. To be specific, the classifier that was trained to distinguish the neural codes associated with low vs. high load of one stimulus type (e.g., the visual configuration of coloured squares) was also able to decode with better-than-chance accuracy the load level of the other stimulus type (e.g., the array of written consonants), and vice versa. This might suggest the existence of common (or, at least, overlapping) attention-based neural codes in the attentional frontoparietal network to support high WM load, regardless of the nature of the material being maintained in WM. However, it is important to note that the existence of a common neural pattern related to the decoding of WM load does not exclude the existence of specific patterns associated with the different types of stimuli to be maintained into WM. Moreover, in this study, the two types of stimuli are based on a visual format, i.e., a configuration of coloured squares vs. written strings of consonants. However, as far as we know, no studies have explored the neural codes associated with the WM maintenance of stimuli belonging to different sensory modalities.

Here, we investigated this issue by using a WM task involving either visual or auditory stimuli (e.g., [37]). We used a delayed match-to-sample task wherein a spatial configuration of visual or auditory stimuli had to be encoded, maintained, and retrieved. Participants navigated across smaller (low WM load) or larger (high WM load) spatial maps containing either visual (colour shades) or auditory (pitch tones) information. Therefore, the processing of the spatial representation was associated with visual or auditory information. During the maintenance phase, the participants were required to keep the spatial configuration of colours or sounds in mind for subsequent retrieval, where only a single colour shade or pitch tone was presented in one of the possible positions. The participants’ task was to recognize whether the test stimulus matched the stimulus presented at encoding in that location or not. Given the increased requirement for selective attention under conditions of higher vs. lower WM load [4,6,8,10,11,12,13,14], we used a conjunction analysis to highlight high-level frontoparietal regions involved either with the maintenance of visual or auditory spatial maps. Within these frontoparietal regions, which are crucial for the maintenance of spatial information [38], we tested whether distinguishable patterns of activation are detectable for the maintenance of visual vs. auditory information using MVPA. The existence of indistinguishable multivariate neural codes for visual vs. auditory stimuli within the frontoparietal network would suggest a supramodal role for these regions regardless of the sensory modality of the stimuli. Conversely, distinguishable patterns of brain activity for auditory vs. visual information would suggest that these high-level regions retain and use a modality-specific code during information maintenance in WM. 

## 2. Materials and Methods

### 2.1. Participants

Fifteen right-handed healthy volunteers took part in the study. Participants were recruited via social media and research bulletin boards and were naive to the purpose of the study. All participants reported being in good health, free of psychotropic or vasoactive medication, and with no past history of psychiatric or neurological disease. They also reported normal or corrected-to-normal vision and normal hearing. Each participant received a cash allowance of 37.50 euros to cover travel costs to the neuroimaging laboratory. One participant was excluded from the data analysis because of within-fMRI-run head movements greater than 3 mm or 3°, leaving fourteen participants for the final analyses (8 females and 6 males, mean age: 24.6 years, ranging from 18 to 43 years, standard deviation: 6.5). The sample size was determined on the basis of a preliminary behavioural pilot study conducted before the main fMRI experiment. Specifically, this preliminary behavioural pilot study was used to establish stimuli and tasks that efficiently allowed us to manipulate WM load and were comparable across the two task modalities: visual and auditory. Twelve right-handed volunteers participated in this pilot study (5 females and 7 males, mean age: 28.6 years, ranging from 21 to 44 years, standard deviation: 9.1 years). All of the participants reported being in good health and were not taking psychotropic or vasoactive medication. They had no history of psychiatric or neurological disease, and had normal or corrected-to-normal vision and hearing. The participants were unaware of the study’s purpose and did not take part in the main fMRI experiment. The stimuli and procedure were the same as those described in Section 2.2 of the main article. In this pilot study, we observed the expected effects on performance accuracy. Specifically, a reliable effect of WM load [F(1, 11) = 6.7, *p* = 0.025, η^2^ = 0.380] was observed, indicating an efficient WM manipulation. Additionally, there was no effect of sensory modality [F(1, 11) < 1, n.s., η^2^ = 0.009], suggesting that participants’ performance in the visual and auditory versions of the task was comparable, thus not biasing the stimulus representation toward one sensory modality or the other. Informed written consent was obtained from each participant. The research described in this article was conducted in adherence with the tenets of the Declaration of Helsinki and approved by the independent Ethics Committee of the IRCCS Santa Lucia Foundation (Prot. CE/PROG.567).

### 2.2. Stimuli and Procedure

**Figure 1:**
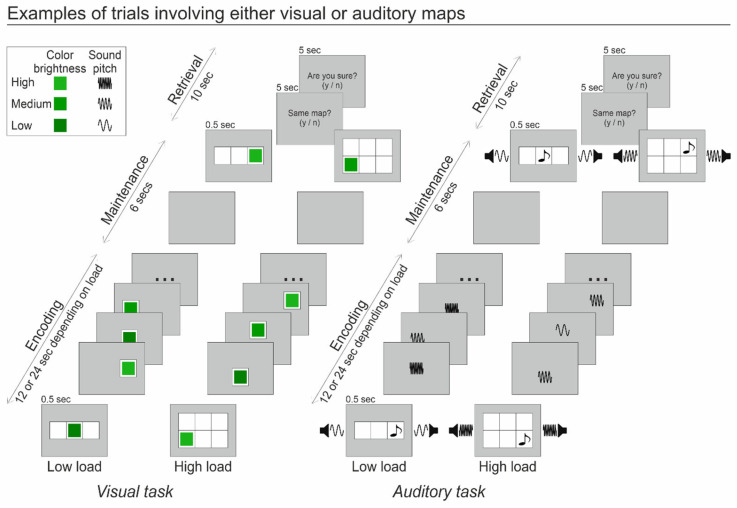
Example of trials involving encoding, maintenance and retrieval of either visual or auditory maps. Each trial began with the presentation of a grid of 3 or 6 cells (depending on WM load) for 0.5 s, including a visual or auditory stimulus placed in a random location. Then, the grid disappeared and participants had to start the navigation moving up, down, left, or right. The participants had 12 or 24 s in which to explore and memorize the maps depending on load (encoding phase). After a maintenance phase of 6 s with a blank background, a test array was presented for 0.5 s (retrieval phase), consisting of a grid that included a stimulus that had either been presented in that position at encoding or not, equiprobably. The participants had 5 s in which to provide a ‘yes’ or ‘no’ response, and a further 5 s for a confidence judgment concerning their response.

The stimuli consisted of either visual or auditory information arranged in spatial maps of variable dimensions (see Figure 1): Low WM load maps consisted of a grid of three possible spatial locations arranged horizontally, while the high WM load maps consisted of a grid of six possible spatial locations arranged in a 2 × 3 matrix. The visual stimuli consisted of squares that could be coloured with one of three different shades of green, either bright (RGB = 26, 179, 26), medium (RGB = 1, 154, 1), or dark (RGB = 2, 127, 1). For the auditory stimuli, we used three pitches instead: high (783.9 Hz), medium (659.2 Hz), and low (523.2 Hz). The volume was subjectively adjusted to be judged as “clearly audible”. Upon entering the fMRI scanner, prior to the start of the main experiment, the participant was presented with the three pitched tones along with the fMRI sequence. The participant was then asked whether the volume of the tones was fine or needed to be increased. The procedure was repeated until the tones were clearly audible to the participant. Every stimulus, either visual or auditory, was presented for 0.5 s.

Each trial began with the presentation of a 3- or 6-location map (depending on the WM load) for 0.5 s. The grid included a visual or an auditory stimulus placed in a random location as a starting point for the map exploration. Note that for auditory maps, the starting spatial position of the sound was marked by a note symbol on the grid (see Figure 1). After 0.5 s, the grid and the stimuli (i.e., the colour shade in visual trials and the note symbol in auditory trials) disappeared, leaving only a grey background, until the participants began the navigation. This was done using four response buttons in a cross arrangement, indicating up, down, left, and right directions, with their right hand. Every time the participants pressed a direction key, a visual (for visual maps) or an auditory (for auditory maps) stimulus was presented in the specific location of the grid where the participants went towards (note that no visual stimulation, i.e., the note symbol, was presented during navigation in auditory trials). Therefore, the participants had the feeling that they could move and explore the whole grid by moving with the direction keys. For instance, in the example trial illustrated in Figure 1 (the low WM load condition of the visual task), the starting point corresponded to the central location of the grid (dark green square). Then, the participant moved on the right location of the grid, where a bright green square was presented. Next, the participants went back to the central location of the grid (where they were presented again with the dark green square) and then moved to the left location where the medium brightness green square was presented, and so on. If participants pressed the arrow key corresponding to a location outside the grid (e.g., the up or down arrow keys in the 3-locations grid), a ‘buzzing’ sound was presented for 0.5 s. Analogously for the auditory maps, the participants explored the specific spatial arrangement of sounds for that trial through the four direction keys. Importantly, no visual signals were provided during the exploration of spatial maps in order to mark the position of the sound. The participants were free to explore each map as they wished with the instruction to keep in mind the specific visual or auditory spatial configuration of that map for the following retrieval test. The participants had 12 s to explore and memorize low WM load maps, and 24 s to explore and memorize the high WM load maps (i.e., 4 s to explore and memorize each cell of the grid irrespective of the dimensions of the map). This approach allowed us to assure that the increased task difficulty in the high-load condition was attributable to an increased amount of information to keep in mind, and not to other factors, such as time constraint. Within each map, each type of stimulus (e.g., the bright green square or the medium pitch sound) could be presented in a maximum of two different spatial locations.

When the time for exploration expired, and after a maintenance period of 6 s with a blank (grey) background, the retrieval phase began. This consisted of the presentation of the grid (as at encoding) with a visual (for visual maps) or auditory (for auditory maps) target stimulus presented in a randomly selected cell. In half of the trials, the target stimulus was the same as the stimulus presented in that location during the exploration of the map, while in the other half of the trials, it was a different stimulus. The test array was presented for 0.5 s, followed by a display asking whether the stimulus belonged to the “same map” as at the encoding phase. The participants had 5 s in which to provide a “yes” or “no” response, pressing the left or right direction key, respectively. The participants were then presented with a second display asking for a confidence judgment (“Are you sure?”) related to their previous response. Once again, they had 5 s in which to provide their response by pressing the left or right direction key for ‘yes’ or ‘no’ response.

During fMRI scanning, the participants completed a total of 80 trials, 40 visual and 40 auditory trials, divided into four blocks, two of which were visual and two auditory. Each block included 20 trials, 10 high and 10 low WM load trials, lasting for approximately 12 min. The order of presentation of the visual and auditory blocks was counterbalanced across participants. Before starting the fMRI session, the participants completed a training session in order to familiarize themselves with the task, including a block of 10 visual and a block of 10 auditory trials, each involving 5 high and 5 low WM load trials.

### 2.3. Magnetic Resonance Imaging

Functional magnetic resonance (MR) images were obtained using a Siemens Allegra systems operating at 3 Tesla and configured for echoplanar imaging (EPI). Radio-frequency transmission and reception were facilitated through the use of a quadrature volume head coil. To mitigate head movement, mild restraint and cushioning measures were employed. The imaging protocol involved acquiring thirty-two slices of functional MR images using blood oxygen level-dependent (BOLD) imaging, with a spatial resolution of 3 × 3 mm, slice thickness of 2.5 mm, 50% distance factor, repetition time of 2.08 s, and a time echo of 30 milliseconds. The entire cortex was covered during the imaging process.

### 2.4. fMRI Data Analysis

For the imaging analysis, we employed SPM 12 (http://www.fil.ion.ucl.ac.uk/spm/, accessed on 3 July 2023), implemented in MATLAB 2010a (The MathWorks Inc., Natick, MA, USA; https://www.mathworks.com/, accessed on 3 July 2023), to conduct data pre-processing and statistical analyses. Each participant underwent four fMRI runs, each consisting of 355 volumes. The initial 4 volumes of each run were discarded, and all images were corrected for head movements. Slice-acquisition delays were rectified using the middle slice as a reference. Subsequently, all images were normalized to the standard SPM12 EPI template, resampled to a 2 mm isotropic voxel size, and spatially smoothed with an isotropic Gaussian kernel of 8 mm full width at half maximum (FWHM). Time series at each voxel for each participant underwent high-pass filtering at 220 s and pre-whitening through the autoregressive model AR(1).

Statistical inference followed a two-step random effects approach: first-level multiple regression models estimated contrasts of interest for each participant, followed by second-level analyses for statistical inference at the group level. Correction for nonsphericity [39] was applied to address potential differences in error variance across conditions. For each participant, the first-level multiple regression model included eight conditions at encoding, eight conditions at maintenance, and eight conditions at retrieval (i.e., twenty-four conditions in total). The eight conditions belonging to each memory phase were given by the crossing of “Task modality” (visual vs. auditory), “Memory load” (high vs. load), and performance (correct vs. incorrect). It is worth noting that “correct” trials included those trials in which the participants provided a correct response at the “same vs. different map” question followed by a confidence judgment (“Yes, I’m sure.”). In fact, with the current two-alternative forced-choice task (same vs. different map), participants may provide quite a few “correct” responses even when failing to recollect the target object location correctly, as a consequence of the 50% chance level. This was the reason why we used correct responses followed by confidence judgments in our main fMRI analysis (for an analogous approach, see [40,41,42]). The eight conditions were modelled at maintenance as miniblocks, time-locked at the offset of the encoding phase, with a duration of 6 s. All predictors were convolved with the SPM12 hemodynamic response function, and the parameters of head movements were included as covariates of no interest.

The primary aim of the present study was to investigate the neural signatures of to-be-maintained auditory and visual stimuli into WM. Accordingly, for each participant, we computed two contrasts that revealed the effect of WM load during WM maintenance, comparing high vs. low load trials that were correctly and confidently reported at retrieval for each task modality. This resulted in 2 contrast images per participant—that is, the effect of “visual WM load” and the effect of “auditory WM load”—each averaged across the two related fMRI runs. The contrast images then underwent the second step, i.e., the group-level analysis, involving a paired *t*-test that modelled the effect of the 2 conditions (auditory and visual WM load), plus the effect of participants. The statistical threshold was set to *p*-FWE-corrected < 0.05 at the cluster level (cluster extent estimated a *p*-uncorrected = 0.001), considering the whole brain as the volume of interest.

This analysis allowed us to assess the existence of differences and commonalities between the neural correlates of WM load in the two sensory modalities. While we did not find differences between auditory and visual WM load at maintenance, we highlighted common regions using a conjunction analysis (see fMRI results below). This showed increased activity as a function of increased WM load in either task modality. Within the region showing a load effect in either task modality, we then conducted MVPAs (see the following section) to determine the existence of distinct (modality-specific) or undistinguishable (supramodal) multivariate patterns of BOLD responses during the maintenance of visual and auditory WM content.

### 2.5. Multi-Voxel Pattern Analysis (MVPA)

After having highlighted high-level regions showing an effect of the WM load during the maintenance phase (see above), we examined whether the neural activity in these regions relied on a modality-specific or supramodal code to represent spatial-related visual and auditory information. For this, we used MVPA, namely, the cross-validated MANOVA (cvMANOVA; http://github.com/allefeld/cvmanova, accessed on 10 July 2023) developed by Allefeld and Haynes [43], to quantify modality-specific differences in the BOLD response patterns in those regions. cvMANOVA has several advantages over more common classified-based “decoding” techniques (e.g., [44,45]), e.g., a continuous instead of a binary classification of pattern differences, a parameter-free analysis based on a probabilistic model of the data (i.e., the multivariate general linear model), and the possibility of obtaining results that can be interpreted in terms of a multivariate effect size (i.e., in terms of explained variance; for more details, see [43,46]).

Before running cvMANOVA, single-participant multiple regression models were re-estimated now using images with a reduced spatial smoothing, using an isotropic Gaussian kernel of 2 mm FWHM [47]. Single-participant models were otherwise identical to those used for the standard fMRI analysis (see previous section), and modelled the maintenance phase through eight conditions, given by the crossing of task modality (visual vs. auditory), memory load (high vs. load), and performance (correct vs. incorrect).

Each single-participant model was used to compute a measure called “pattern distinctness” (D) with cvMANOVA, which directly quantifies the degree to which multivariate distributions of BOLD response patterns in each condition differ from each other within each of the high-level regions showing a main effect of WM load. For this, we run within each region of interest (ROI), i.e., the regions showing an effect of WM load during the maintenance phase, three contrasts directly comparing the multivariate BOLD response pattern at maintenance related to: (1) overall visual vs. auditory information (i.e., including all trials, both low and high WM load); (2) visual vs. auditory information at low WM load; (3) visual vs. auditory information at high WM load. MarsBar 0.41 (“MARSeille Boîte À Région d’Intérêt” SPM toolbox) was used to build spherical ROIs with a radius of 8 mm (matching the FWHM of the smoothing filter of the standard analysis) centred on the peak of the regions showing a main effect of the WM load. This procedure allowed us to extract a pattern distinctness value (D) for each participant and contrast within each ROI. If each of the tested contrasts were to elicit the same multivariate response in a given ROI, each pattern distinctness value D would be on average equal to zero, while different multivariate responses would lead to an average D greater than zero. For this reason, we tested whether the mean value of pattern distinctness (D) between visual and auditory trials was significantly larger than zero within each ROI, using one-tailed one-sample *t*-tests (see, for a similar approach, [46]). 

### 2.6. Statistical Approach

All statistical analyses were performed using JASP 0.14.1.0 (https://jasp-stats.org/, accessed on 15 June 2023). Alongside frequentist analyses, Bayesian analyses were conducted to ascertain the strength of evidence for each outcome [48], in accordance with the subsequent criteria: BF^10^ < 1, indicating no evidence; BF^10^ ranging between 1 and 3, indicating anecdotal evidence; BF^10^ ranging between 3 and 10, indicating substantial evidence; BF^10^ ranging between 10 and 30, indicating strong evidence; BF^10^ ranging between 30 and 100, indicating very strong evidence; and BF^10^ > 100, indicating decisive evidence. For the Bayesian version of the frequentist ANOVA, we performed a Bayesian repeated measure ANOVA, as implemented in JASP, with a uniform model prior and the following coefficient priors: r scale for fixed effect = 0.5, r scale for random effect = 1. On the contrary, for the Bayesian version of the one-sample *t*-test, we performed Bayesian one-sample *t*-tests, as implemented in JASP, with a Cauchy prior distribution centred around zero and with scale of r = 0.707. 

## 3. Results

### 3.1. Behavioural Data

**Figure 2:**
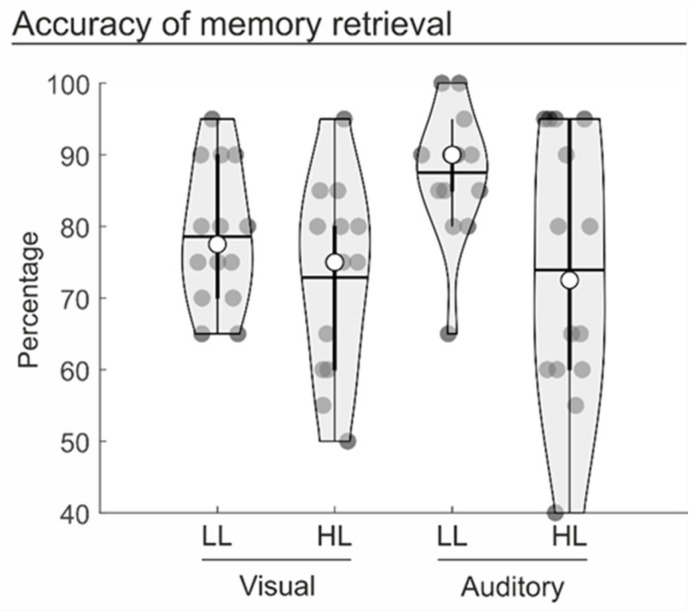
Violin plots displaying the participants’ accuracy of memory retrieval in the different experimental conditions. The dark circles represent the individual performance; the median and mean of the distribution are represented by the white circle and the horizontal bar, respectively. LL = low load trials; HL = high load trials.

Memory accuracy was analysed via a repeated-measure analysis of variance (ANOVA) (see Figure 2). The ANOVA included the within-participants factors of Task modality (visual vs. auditory) and Memory load (high vs. low). The ANOVA revealed a main effect of memory load [F(1, 13) = 13.8, *p* = 0.003, η^2^ = 0.516, BF^10^ = 27.27], indicating a significant decrease in accuracy for high (73.4%), as compared to low (83.0%), load spatial maps. The ANOVA did not reveal any significant differences between the two task modalities [F(1, 13) = 3.3, *p* = 0.092, η^2^ = 0.203, BF^10^ = 1.03], with a similar accuracy in both the visual (75.7%) and auditory (80.7%) tasks. Analogously, there was no interaction between the two factors [F(1, 13) = 1.9, *p* = 0.196, η^2^ = 0.125, BF^10^ = 0.76]. These results indicate a similar effect of WM load between the two task modalities.

### 3.2. fMRI Data

**Figure 3:**
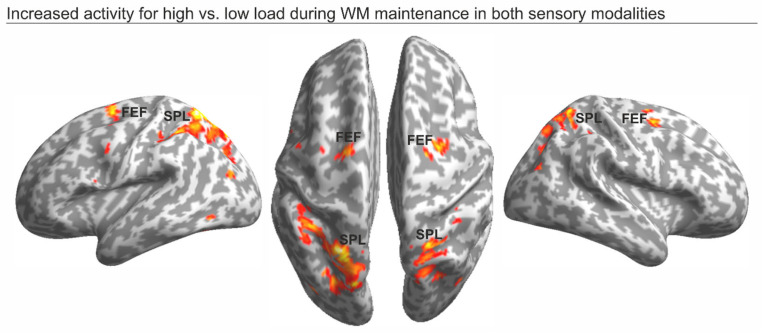
Dorsal frontoparietal regions showing increased activity during the maintenance of high vs. low load maps in either modality (i.e., the conjunction analysis), overlaid on an inflated template (cf. Table 1).

First, we highlighted the regions involved with the maintenance of high vs. low WM load trials in either modality using a conjunction analysis. This revealed widespread activity along the dorsal frontoparietal network (e.g., see [49]), peaking on the left and right frontal eye field (FEF) anteriorly, and on the left and right superior parietal lobule (SPL) posteriorly (see Figure 3 and Table 1). These regions showed a selective increase of activity when participants were involved with the maintenance of high-load maps, as compared to low-load maps, in either the visual or auditory modality.

**Figure 4:**
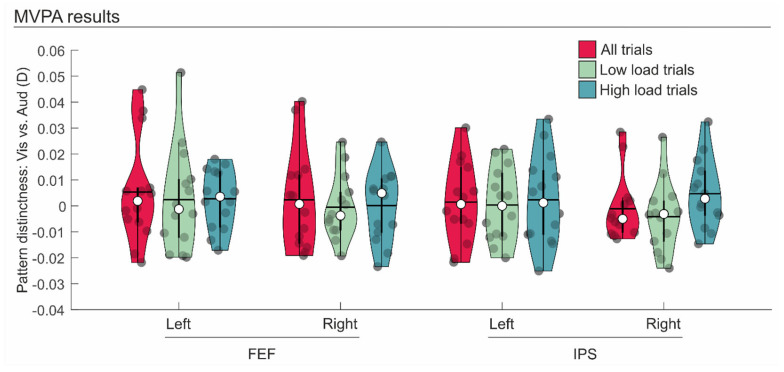
Violin plots displaying the results of the multi-voxel pattern analysis (MVPA) in terms of the mean pattern distinctness (D value) of visual vs. auditory maps for each region of interest. In red, the MVPA that included all trials (both low and high load) are displayed; in light green, the MVPA that included only low load trials was displayed; in light blue, the MVPA that included only high load trials was displayed (cf. Table 2).

Within these regions, we conducted MVPAs using cvMANOVA, with the aim of quantifying the pattern of distinctness of visual vs. auditory neural codes during WM maintenance (see Figure 4 and Table 2). All the MVPAs failed to reveal any distinctness between the patterns associated with visual vs. auditory information across the four ROIs. This was evidenced by patterns that were not significantly larger than 0 (all *t*s < 1.327, all *p*s > 0.104; see Table 2 and Figure 4). This finding held true when considering all visual vs. auditory trials (i.e., both low and high WM load), only visual vs. auditory low WM load trials, or only visual vs. auditory high WM load trials. Consistently, the Bayesian analysis revealed BF^10^ values that were lower than 1 for all contrasts, thus further confirming that there was no evidence for distinguishable brain patterns related to the maintenance of visual vs. auditory stimuli in the selected ROIs (see Table 2).

## 4. Discussion

The aim of the current study was to investigate whether high-level frontoparietal regions make use of common (“supramodal”) or distinct (“modality-specific”) neural codes during the maintenance of visual vs. auditory spatial information. During fMRI scanning, participants explored high- or low-load spatial maps, encoding the spatial location of different colours (visual maps) or pitches (auditory maps). During maintenance, participants were asked to retain the spatial configuration of the previously explored visual or auditory stimuli for a subsequent memory test. This involved the presentation of a single stimulus (a colour for visual trials, and the pitch of a sound for auditory trials) at a given location on the map. The participants’ task was to judge whether the test stimulus (colour or pitch) matched the stimulus at the corresponding location on the map they had studied during encoding. They then had to make a confidence judgement. 

Behavioural results revealed impaired performance (i.e., lower accuracy) following high-load versus low-load trials, demonstrating an effective manipulation of WM load with the current experimental design. They also showed that participants performed similarly on both the visual and auditory versions of the task (i.e., there was no main effect of sensory modality, nor any interaction between WM load and sensory modality). This was important to reduce the possibility that the observed neural representations (see MVPA analysis) were biased towards either visual or auditory information.

At the neuroimaging level, we found that the maintenance of the increased WM load, either in the visual or auditory domain, was supported by increased activity along the dorsal frontoparietal network, peaking at the FEF and SPL, bilaterally. Increased activation within the dorsal frontoparietal network as a function of increased WM load has previously been described during WM tasks (e.g., see [6,50,51]), but also during selective attention tasks (see [22], for a meta-analysis). Consistent with this view, we found that the more demanding the WM task was (i.e., high-load maps), the more active the dorsal frontoparietal regions were, thus supporting successful WM maintenance, regardless of the sensory modality in which the task was performed. However, the existence of common areas associated to the maintenance of high-load visual or auditory spatial maps does not automatically exclude the existence of specific brain signatures for visual vs. auditory information.

Here, we tested this possibility using MVPAs, which revealed no distinguishable patterns of distinctness for visual vs. auditory neural codes during WM maintenance, in any of the considered high-level frontoparietal regions—that is, those regions involved in the maintenance of visual or auditory spatial maps (i.e., the SPL and the FEF, bilaterally). These results support the existence of a supramodal neural code associated with the WM maintenance of visual or auditory spatial stimuli under conditions of high WM load. These findings are consistent with a large body of literature supporting a supramodal attentional role of frontoparietal regions (e.g., [24,25,26,27,28,29]). Moreover, they complement and extend the previous findings of Majerus and colleagues [20] on the supramodal role of these regions in representing the load during WM maintenance. Indeed, whereas Majerus and colleagues found a common representation of the load level in the frontoparietal network, as revealed by better-than-chance decoding of the low vs. high load across two stimulus typologies (i.e., written vs. graphical visual stimuli), here we found that the frontoparietal network involved in high-load maintenance does not retain and use a modality-specific neural code for processing visual vs. auditory information during the maintenance phase. Importantly, the current results do not contradict, but instead extend, previous findings suggesting modality-specific neural codes during WM n-back tasks in frontoparietal regions (see, e.g., [33,34]), as these modality-specific patterns were observed during ongoing sensory stimulation (i.e., during visual or auditory stimulation in the encoding phase). Here, in contrast, the patterns of neural codes were measured and analysed (with MVPA) during the maintenance phase, providing support for an indistinguishable stimulus-modality representation in the absence of sensory stimulation.

Following this line of reasoning, these findings also extend our knowledge of the role played by frontoparietal regions in the representation of specific WM content. There is a large corpus of literature focusing on where in the brain WM contents are stored during the maintenance phase (e.g., [52]; for reviews, see, [53,54]). Most of these studies demonstrated persistent and distributed representation of WM contents within sensory regions [55,56,57]. However, some of these also found the representation of the maintained information within the attentional frontoparietal network, such as colour patterns [58,59], stimulus positions [56,60], and motion features [61]. Furthermore, in both frontal and parietal regions, object identities associated with spatial locations [62] and line orientations [63] could be decoded. Here, we contribute to this literature by highlighting the WM representation of visual and auditory spatial information in frontoparietal regions, providing support for a common, supramodal neural code associated with the maintenance of high-load information.

An important limitation of the present study is the lack of control over the strategy that participants may have used to retain visual and auditory information, which could also involve verbal/phonological recoding of spatial information (e.g., [64,65]). Moreover, although the current sample size was found to be sufficient to observe WM load effects, it may be important to increase the sample size in future studies to further confirm the lack of differences between visual and auditory information. Relatedly, it could be argued that the lack of differences between the neural representation of visual and auditory information during WM maintenance may be due to a limited sensitivity of the specific MVPA algorithm used here (namely, cvMANOVA). Therefore, it is recommended that future research uses a larger sample size and alternative MVPA algorithms on similar task designs to gain a more comprehensive understanding of this research area. Notwithstanding the issues that could be clarified by future studies, the present findings indicate no discernible differences in the multivoxel patterns associated with the WM maintenance of visual and auditory information. 

## 5. Conclusions

The present study investigated the neural representation of visual and auditory spatial information within the dorsal frontoparietal network during WM maintenance, suggesting the existence of a supramodal code associated with the retention of either visual or auditory spatial configurations. Future research will need to understand the extent to which the observed common neural code depends on the specific pair of sensory modalities tested here, or on the specific task we used, or whether these findings could be generalized to other sensory modalities and WM tasks.

## Figures and Tables

**Table 1:** *p*-FWE-corrected values, cluster size, peak *t*-value, and peak MNI coordinates (*x*, *y*, *z*) for the brain regions showing significant activation during the maintenance of high vs. low load maps in either sensory modality.

Brain Region	Cluster *p*-FWE-Corr	Cluster Size (k)	Peak *t*-Value	Peak Coordinates X Y Z
Left FEF	0.018	192	7.22	−22 −4 58
Right FEF	0.016	198	6.32	26 −2 48
Left SPL	<0.001	1979	10.77	−22 −62 62
Right SPL	<0.001	595	8.69	22 −62 64

Note: FEF: frontal eye-fields; SPL: superior parietal lobule.

**Table 2:** One-tailed one-sample *t*-value, *p*-value, Cohen’s d, and Bayes factor (BF^10^) values for each region of interest in the MVPAs contrasting the maintenance of visual vs. auditory maps including all trials (i.e., both low and high load trials), only low load trials, and only high load trials.

Contrast	Brain Region	*t*(13)	*p*-Value	Cohen’s d	BF^10^
Visual vs. Auditory: all trials	Left FEF	0.991	0.170	0.265	0.668
	Right FEF	0.458	0.327	0.122	0.392
	Left SPL	0.350	0.366	0.094	0.357
	Right SPL	−0.325	0.625	−0.087	0.216
Visual vs. Auditory: only low load trials	Left FEF	0.453	0.329	0.121	0.391
	Right FEF	−0.165	0.564	−0.044	0.240
	Left SPL	0.097	0.462	0.026	0.290
	Right SPL	−1.168	0.868	−0.312	0.141
Visual vs. Auditory: only high load trials	Left FEF	0.898	0.193	0.240	0.604
	Right FEF	0.035	0.486	0.009	0.277
	Left SPL	0.501	0.312	0.134	0.408
	Right SPL	1.326	0.104	0.354	0.986

Note: FEF: frontal eye-fields; SPL: superior parietal lobule.

## Data Availability

The dataset generated and analysed in the current study is not publicly available due to ethical restrictions, but it is available from the corresponding author on reasonable request.
